# Four Meroterpenoids with Novel Aminoglycoside Moiety from the Basidiomycete *Clitocybe clavipes* with Cytotoxic Activity

**DOI:** 10.3390/molecules28145456

**Published:** 2023-07-17

**Authors:** Zhonghao Sun, Yongben Ma, Jiawen Zhang, Guoxu Ma, Haifeng Wu, Leiling Shi, Zhaocui Sun, Xudong Xu

**Affiliations:** 1 Institute of Medicinal Plant Development, Chinese Academy of Medical Sciences & Peking Union Medical College, Beijing 100193, China; sun_zhonghao@126.com (Z.S.);; 2 School of Pharmaceutical Sciences, Hebei University of Chinese Medicine, Shijiazhuang 050091, China; 3 Xinjiang Institute of Chinese and Ethnic Medicine, Urumqi 830002, China

**Keywords:** meroterpenoid, clavilactone, *Clitocybe clavipes*, cytotoxicity

## Abstract

Four new meroterpenoids, Clavilactone M-P, possessing novel aminoglycoside moiety (**1**–**4**) and a 10-membered carbocycle fused with an α,β-epoxy-γ-lactone, were isolated from *Clitocybe clavipes*, a basidiomycete. Their structures with absolute configurations were determined by extensive analysis of their spectroscopic data, and the ECD method. All the isolated compounds (**1**–**4**) were evaluated for their antitumor activity against three human cancer cell lines using the MTT assay. Compound **1** and **2** exhibited a significant suppression of cell viability in the Hela (IC_50_ = 22.8 and 19.7 μM) cell line.

## 1. Introduction

Meroterpenoids are a hybrid class of natural products with structural of a terpenoid moiety and a complex molecular scaffold [1], which were discovered in a variety of organisms, such as plants, marine organisms, and fungi [2,3,4]. Clavilactones are kinds of special meroterpenoids usually bearing a benzo-fused 10-membered carbocycle units, which were first isolated by Nasini in 1994 [5]. These kinds of compounds displayed significant biological activities, including antibacterial [6], antifungal [7], anti-tumor [8,9,10], myocardial protection [11], anti-inflammatory [12,13], and neuroprotective [14] activities. In particular, ‘clavilactone’ stands for a novel class of tyrosine kinase inhibitors [15], and clavilactone D was shown to inhibit epidermal growth factor receptor tyrosine kinase, with an IC_50_ value of 5.5 µM [8,16]. 

**Figure 1:**
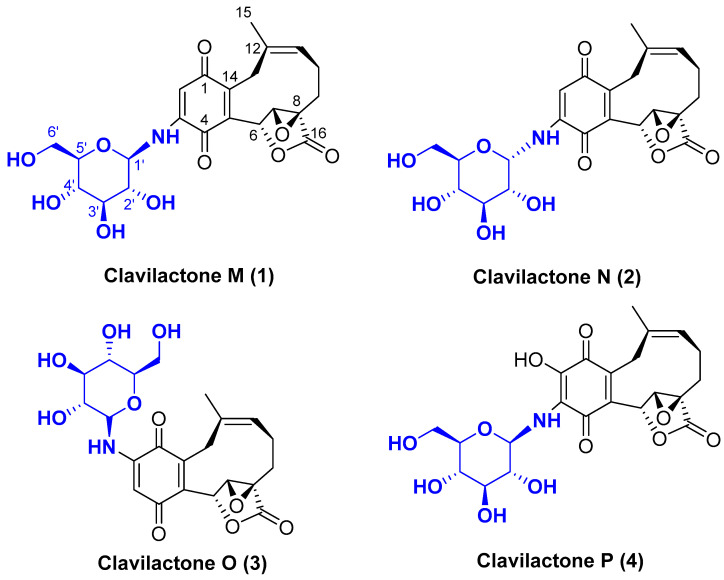
Structures of compounds **1**–**4**.

So far, no more than 40 naturally occurring meroterpenoids containing benzo-fused 10-membered carbocycle fragments have been found from natural sources. Our group has long been interested in this type of structure and discovered several kinds of novel clavilactones from *Clitocybe clavipes*. For instance, clavilactones G-K were isolated, which all possessed a 10-membered carbocycle connected to a hydroquinone with moderate cytotoxic activities [17,18]. Previously, we have reported the isolation of two kinds of novel nitrogen-containing meroterpenoids, clavipyrrine A [19] and clavipines A–C [20], from the basidiomycete *Clitocybeclavipes*. These unique structures with promising anti-tumor activity led to our ongoing investigation of nitrogenous meroterpenoids. As a result, four new aminoglycoside meroterpenoids, clavilactone M-P (**1–4**) (Figure 1), were obtained. To the best of our knowledge, this is the first report on aminoglycoside meroterpenoids in nature. Herein, their isolation and structural elucidation, as well as their cytotoxic activities, are described.

## 2. Results

Compound **1**, obtained as a red powder, was determined to have the molecular formula C_22_H_25_NO_10_ by the positive HR-ESI-MS ion peak at *m*/*z* 464.1552 [M+H]^+^ (calculated as 464.1557, C_22_H_26_NO_10_), suggesting the presence of 10 indices of hydrogen deficiency (IHDs). The IR spectrum of **1** showed unambiguous absorption bands for the hydroxyl or amino groups (3340 cm^−1^). The ^1^H-NMR spectrum (Table 1) of **1** suggested the presence of one aromatic proton and one olefinic methine (δ_H_ 5.94(1H, s, H-2), 5.98(1H, s H-6)), four aliphatic methylenes (δ_H_ 1.3(1H, m, H-9b), 2.60(1H, d, *J* = 13.2 Hz, H-9a), 2.18(1H, m, H-10b), 2.35(1H, m, H-10a), 2.84(1H, d, *J* = 12 Hz, H-13b), 3.67(1H, d, *J* = 12 Hz, H-13a), 3.68(1H, dd, *J* = 2.4, 12.0 Hz, H-1′a), 3.86(1H, dd, *J* = 5.4, 12.0 Hz, H-1′b)), and one two-methyl group (δ_H_ 1.53(3H, s, H-15)). The ^13^C NMR spectra, with the aid of the HSQC spectrum, revealed 22 carbon signals which were attributed to one methyl, four methylenes, two olefinic methines, five quaternary carbons (four olefinic quaternary carbons), three carbonyls, and seven oxygenated methines. The comprehensive analysis of ^1^H and ^13^C NMR data suggested the existence of a quinone skeleton (δ_C_: 186.1, C; 183.0, C; 103.0, CH; 148.1, C; 151.9, C; 135.6) in compound **1**. Two olefinic carbons (δ_C_ 124.9, 136.9) and the methyl carbon (δ_C_ 23.4) were assigned to the methyl substituted trisunbstituted -C=C- group.

**Figure 2:**
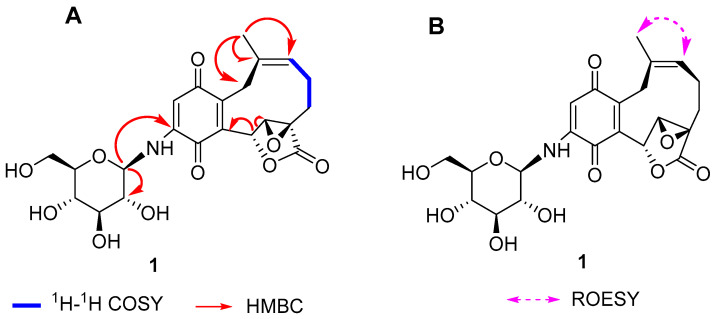
(**A**) Key ^1^H–^1^H COSY and HMBC correlations of compounds **1**. (**B**) ROESY correlations of **1**.

The ^1^H-^1^H COSY spin fragment of H-9/H-10/H-11 (Figure 2A), along with the HMBC correlations from H-15(δ_H_ 1.53, s) to C-11(δC 124.9), C-12(δC 136.9), and C-13(δC 27.8) (Figure 2A) established the group, -CH_2_C(CH_3_)=CHCH_2_CH_2_-. Furthermore, ^13^C-APT NMR signals of 74.1(C-6), 64.2(C-7), 61.8(C-8), and 172.8(C-16) indicated the presence of an α, β-epoxy ɤ-lactone moiety in **1** [21], which was established by the HMBC correlations from H-6(5.98, s) to C-7(δ_C_ 64.2) (Figure 2). The HMBC correlations from H-6(δ_H_ 5.98, s) to C-5(δC 151.9) confirmed that this fragment was attached to C-5. 

An anomeric signal was obviously observed at δ_H_ 4.52(1H, d, 8.4) and δ_C_ 84.4(C-1′), which combined with one oxygenated methylene carbon (δ_C_ 62.7) and four oxygenated methine carbons (δ_C_ 79.6, 78.7, 74.1, 71.5) indicated the existence of a glucose fragment in the molecule. In addition, the HMBC correlations of H-1′ to C-3(δ_C_ 148.1) and C-2′ revealed the glucose fragment was attached to the C-3 position (Figure 2). The acid hydrolysis of **1** liberated the D-glucopyranose, which was in agreement with the remaining ^1^H, ^13^C NMR data and HMBC correlations. With overall consideration of these signals, the planar structure of compound **1** was considered an unusual *β*-D-aminoglycoside meroterpenoid containing a benzoquinone fused to a ten-membered carbocycle with α, β-epoxy-γ-lacton. 

The relative configuration of **1** was revealed by the proton coupling and the NOE data. H-6(δ_H_ 6.76, s) and H-7(δ_H_ 4.08, s) protons form a dihedral angle of about 90° as there is no vicinal coupling between the two adjacent protons. This suggests that the relative configuration of C-6, C-7, and C-8 is either 6*R*, 7*R*, 8*R* or 6*S*, 7*S*, 8*S*. Meanwhile, the *Z* geometry of C-11/C-12 olefin was identified by the key ROESY correlations of H-11/H-15 labelled in Figure 2B. 

**Figure 3:**
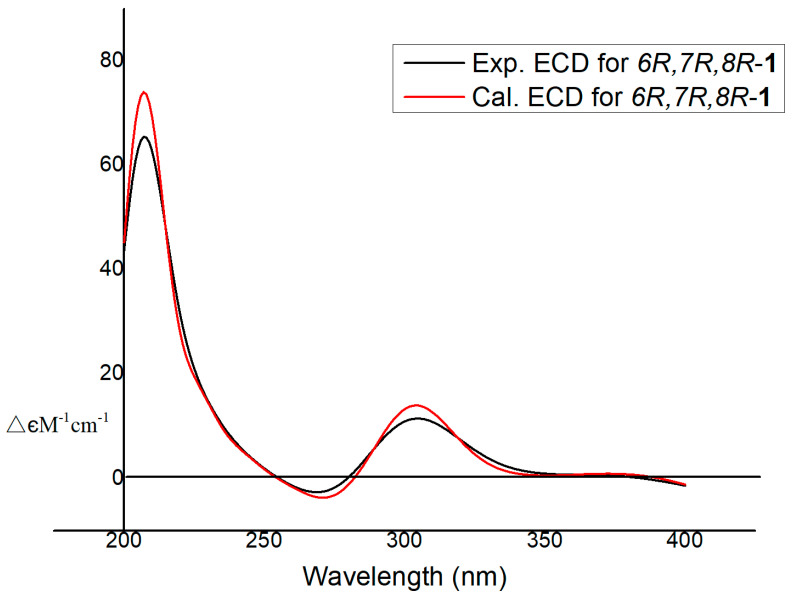
Calculated and experimental electronic circular dichroism (ECD) spectra of **1** in methanol.

The ECD spectra were calculated using density functional theory (DFT) at the APFD/6-311+g (2d, p) level. The aglycone of **1** was used to conduct the ECD experiment. The comparison of the theoretically calculated and experimental ECD curves showed that the calculated ECD spectrum of (6*R*,7*R*,8*R*)-**1** fitted better with the experimental one than the ECD spectrum of (6*S*,7*S*,8*S*)-**1** (Figure 3). Finally, the stereoabsolute configurations of the C-6, C-7, and C-8 positions of compound 1 are determined to be 6*R*, 7*R*, and 8*R*, and the compound was named Clavilactone M.

**Figure 4:**
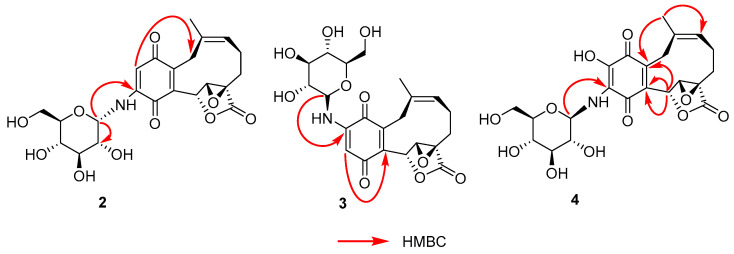
Key HMBC correlations for compounds **2**–**4**.

Compound **2** was obtained as a red powder. Its molecular formula was determined to be C_22_H_25_NO_10_4 according to its HRESIMS at *m*/*z* 486.1382 [M + Na]^+^ (calculated for 486.1376, C_22_H_25_NO_10_Na), indicative of ten IHDs. The quinone unit (δ_C_: 185.8, C; 182.8, C; 103.8, CH; 147.5, C; 151.8, C; 135.5) and glucose fragment (δ_C_: 82.3, CH; 74.0, CH; 78.6, CH; 71.6, CH; 79.4, CH; 62.3, CH_2_) as well as other carbon signals (173.6, 136.7, 124.7, 64.1, 61.6, 27.6, 25.5, 23.6, 23.1) were largely identical with that of compound **1** (Table 1). The only difference was the anomeric proton C-1′ shifted to δ_H_ 5.11 compared with compound **1** at δ_H_ 4.52, and the coupling constant of the anomeric proton became 4.8 Hz, which suggested that the sugar moiety in **2** was α-D-N-glucose. The sugar unit was identified as D-glucose by TLC in comparison with authentic D-sugar (visualization with ethanol 5%H_2_SO_4_ spraying) followed by gas chromatography. Meanwhile, the HMBC correlations of H-2 to C-13(δC 27.6) and H-1′ to C-3(δC 147.5)/C-2′(δC 74.0) revealed the α-D-N-glucose was contained in the molecular and the amino was substituted at the C-3 position (Figure 4). The planar structure of **2** was shown in Figure 1. On the basis of biogenetic consideration, the absolute configuration at the C-6, C-7, and C-8 positions of **2** was proposed to be same as that of **1**. Meanwhile, the experimental ECD spectra of their aglycones showed the identical tendency (supplementary Figures S29). Thus, the absolute configuration of **2** was identified as 6*R*, 7*R*, 8*R*, and the compound was given the name Clavilactone N.

Compound **3**, a red powder, had the molecular formula C_22_H_25_NO_10_ as established by the HR-MS-ESI (*m*/*z*: [M+Na]^+^ 486.1388, calculated as 486.1376, C_22_H_25_NO_10_Na), indicating ten indices of hydrogen deficiency. Compared with compound **1**, the ^1^H-NMR and ^13^C-NMR spectra data of **3** also displayed a quinine moiety (δ_C_ 186.1, 183.0, 102.7, 148.1, 146.1, 136.4), a *β*-D-glucose fragment (δC 84.3, 79.6, 78.8, 74.1, 71.5, 62.7), and other signals similar to that of **1** (δC 173.8, 139.0, 125.2, 74.0, 64.3, 61.9, 27.5, 25.7, 23.8, 23.2). However, the HMBC correlations of H-1′ to C-2(δC 148.1) and H-3 to C-5(δC 146.1) revealed the glucose was substituted at the C-2 position (Figure 4). The acid hydrolysis of **3** liberated the D-glucopyranose; the absolute configurations of the sugars were also determined by gas chromatography as *β*-D-glucose. Finally, the planar structure of **3** was established as shown in Figure 1. In the ECD experiment, the trend experimental data of aglycone from **3** was consistent with that of **1** (Supplementary Figure S29). From the perspective of the biosynthetic pathway and ECD experiment, the absolute configuration of **3** was determined to be 6*R*, 7*R*, 8*R*, and the compound was given the name Clavilactone O. 

Compound **4** obtained as a red powder, was determined to be with the molecular formula of C_22_H_25_NO_11_ by the positive HR-ESI-MS ion peak at *m*/*z* 502.1335 [M+Na]^+^(calculated as 502.1325), corresponding to 10 degrees of unsaturation. Overall consideration of ^1^H- and ^13^C-APT NMR spectral data (Table 2) suggested that compound **4** was a clavipine-type meroterpenoid with an α, β-epoxy-ɤ-lactone ring, similar to Clavilactone D (δ_C_ 183.9, 181.9, 172.1, 149.2, 146.9, 135.1, 134.1, 133.3, 123.4, 72.7, 62.9, 60.6, 26.6, 24.3, 22.5, 21.8). In detail, the two carbonyl carbons (δ_C_ 183.9, 181.9) and the four olefinic carbons (δ_C_ 149.2, 146.9, 133.3, 135.1) indicated the presence of a quinone skeleton. Furthermore, ^13^C-APT NMR signals of 72.7(C-6), 62.9(C-7), 60.6(C-8), and 172.1(C-16) and their relevant HSQC correlations indicated an α,β-epoxy ɤ-lactone moiety contained in the structure, which was also proved by HMBC between H-7(4.5, s) and C-6(72.7) (Figure 4). The only difference was a downfield shift carbon at δ_C_ 149.2, which suggested the carbon was substituted by oxygen. The anomeric signal δ_C_ 83.1(C-1′), oxygenated methylene carbon (δ_C_ 61.2) and four oxygenated methine carbons (δ_C_ 78.6, 77.2, 72.1, 70.2) indicated the existence of a glucose fragment. The acid hydrolysis of **4** confirmed the sugar moiety as *β*-D-glucopyranose. The anomeric proton H-1′(4.39, t, 7.2) and its HMBC correlations to C-3(δ_C_ 146.9) revealed the attachment of a *β*-D-aminoglycoside moiety at the C-3 position. The four olefinic carbons were assigned by the HMBC correlations between H-6 (δ_H_ 5.87) and C-5(133.3), C-14(135.1) (Figure 4). The HMBC correlations between H-15(δH, 1.47, 3H, s) and C-14(135.1) also confirmed the assignment of olefinic carbons (Figure 4). Accordingly, the entire structure of compound **4** is elucidated in Figure 4. The absolute configuration of **4** was established by comparing the experimental curve of ECD with that of compound **1** (Supplementary Figure S29). The ECD spectrum of their aglycones matched well, which confirmed the 6*R*,7*R*,8*R* configuration, and the compound was given the name Clavilactone P.

In addition, the antitumor activity was measured by IC_50_ value against three human cancer cells (Hela, SGC-7901, and SHG-44) using the MTT assay [22]. Cisplatin was used as a standard for comparison. The antitumor effects are displayed in Table 3. Compound **1** and **2** exhibited moderate cytotoxic activity against Hela cell lines, with IC_50_ values of 22.8 and 19.7 µM, respectively.

## 3. Discussion

In summary, four meroterpenoids with a novel aminoglycoside moiety were isolated for the first time from the fungus *C. clavipes*, and they represent the first group of aminoglycoside meroterpenoids possessing a 10-membered carbocycle fusing α,β-epoxy-γ-lactone. The novel aminoglycoside is a rare in nature, and this kind of nitrogenous meroterpenoid not only enriched the structural diversity, but also provided potent activity. Meanwhile, the glycosylation of natural products and drugs can often effectively change their physical and chemical activities, so natural glycoside compounds have great advantages in drug discovery, for example, the modification of digitoxigenin by glycosidation with neogluco/xylosides revealed sugar amine regiochemistry and had a dramatic effect upon anti-tumor activity [23]. The screening of cytotoxic activity proved that **1** and **2** exhibited a significant suppression of cell viability in the Hela (IC_50_ = 22.8 and 19.7 μM) cell line. The relationship between glycosidation and the cytotoxic activity of this scaffold need to be further studied. 

## 4. Materials and Methods

### 4.1. General Experimental Procedures

NMR spectra were obtained with a Bruker AV 600 NMR spectrometer (chemical shift are presented as δ values with TMS as the internal standard) (Bruker, Billerica, Germany). Abbreviations are as follows: s (singlet), d (doublet), dd (doublet of doublet) t (triplet), q (quartet), m (multiplet), bs (broad singlet). Chemical shifts (δ) are given in ppm relative to solvent residual peak (CD_3_OD, δ = 3.3 ppm, DMSO-d6, δ = 2.5 ppm) as external standard. High resolution mass spectra (HR-ESI-MS) was conducted with ThermoFisher Scientific LTQ-Orbitrap XL spectrometer (Waters, Milford, MA, USA). UV and IR data were obtained using a Shimadzu UV2550 spectrophotometer and a FTIR-8400S spectrometer (Shimadzu, Kyoto, Japan), respectively. Precoated silica gel GF254 plates (Zhi Fu Huang Wu Pilot Plant of Silica Gel Development, Yantai, China) were needed for TLC. Semi-preparative HPLC was conducted on an analytic LC equipped with a pump of P230 and a DAD detector of 230+ (Ellte, Dalian, China) with a C18 ODS-A (5 µm, YMC, Kyoto, Japan). Column chromatography used silica gel columns (200–300 mesh, Qingdao Marine Chemical plant, Qingdao, China). All solvents used were of analytical grade (Beijing Chemical Plant, Beijing, China).

### 4.2. Fungal Material

The fungal strain of *Clitocybeclavipes* (CBS 126.44) was purchased from Central Bureau voor Schimmelcultures (Baarn). The strain was preserved at the key laboratory of the Institute of Medicinal Plant Development (No. 20180742). The fungus *C. clavipes* was cultured on YMGA (yeast:malt:glucose:agar, 10:10:30:15 g L^−1^) medium and grown in 600 Petri dishes (90 mm × 15 mm) for 25 days at 28 °C.

### 4.3. Extraction and Isolation 

EtOAc extracts (7.5 g) were subjected to silica-gel (200–300 mesh) column chromatography (CC) with two gradient systems (ether/EtOAc 50:1, 30:1, 10:1, 5:1, 1:1; CH_2_Cl_2_/MeOH 20:1, 10:1, 5:1, 1:1, 0:1 *v*/*v*) to give 8 fractions (F1–F10). F9 was purified by semi-preparative HPLC (CH_3_CN/H_2_O 60:40, *v*/*v*) to yield **1** (6 mg, *t*_R_ = 16.5 min), **2** (3 mg, *t*_R_ = 17.1 min) and **3** (4 mg, *t*_R_ = 18.1 min). F10 were subjected to C-18 reversed-phase (RP) silica-gel CC using MeOH/H_2_O in a linear gradient (30:70, 45:55, 60:40, 80:20, 100:0, *v*/*v*) to obtain 5 fractions (F10-1–F10-5). F10-1 was purified by semi-preparative HPLC with CH_3_CN-H_2_O as mobile phase (55:45, *v*/*v*), to give **4** (4 mg, *t*_R_ = 12.4 min).

The structures of compounds **1**–**4** were determined by HRESIMS, UV, IR, 1D and 2D-NMR spectra.

Clavilactone M (**1**), red powder; UV(MeOH) λ_max_ (log ε) 204(3.54), 281(3.20) nm; IR(KBr) *v*_max_ 3440, 3349, 2947, 1778, 1641, 1597, 1420, 1336, 1231, 1149, 1071, 821 cm^−1^. ^1^H and ^13^C-NMR data see Table 1; (+)HRESIMS *m*/*z* 464.1552 [M+H]^+^(calculated as 464.1557).

Clavilactone N (**2**), red powder; UV(MeOH) λ_max_ (log ε) 205(3.81), 280(3.41) nm; IR(KBr) *v*_max_ 3430, 3342, 2951, 1780, 1645, 1592, 1345, 1228, 1150, 1078, 830 cm^−1^. ^1^H and ^13^C-NMR data see Table 1; (+)HRESIMS *m*/*z* 486.1382 [M+Na]^+^(calculated as 486.1376).

Clavilactone P (**3**), red powder; UV(MeOH) λ_max_ (log ε) 205(3.58), 281(3.27) nm; IR(KBr) *v*_max_ 3435, 3344, 2943, 1776, 1643, 1593, 1422, 1335, 1245, 1148, 1075, 831 cm^−1^. For ^1^H and ^13^C-NMR data, see Table 1; (+)HRESIMS *m*/*z* 486.1388 [M+Na]^+^(calculated as 486.1376).

Clavilactone Q (**4**), red powder; UV(MeOH) λ_max_ (log ε) 210(3.50), 288(3.11) nm; IR(KBr) *v*_max_ 3351, 2944, 1775, 1645, 1601, 1338, 1230, 1149, 1070, 825 cm^−1^. ^1^H and ^13^C-NMR data see Table 1; (+)HRESIMS *m*/*z* 502.1335 [M+Na]^+^(calculated as 502.1325).

### 4.4. Acid Hydrolysis and Monosaccharide Identification

The compounds (2–3 mg) were dissolved in 2 mL of 0.5 M HCl in MeOH solution for 1 h and diluted with water (2 mL). The resulting solution was neutralized with aq. NaOH; after the removal of the solvent, 4 mL of water was added to the residue and it was extracted three times with CH_2_Cl_2_. Sugars were analyzed by TLC and compared with authentic samples of D-sugar. Furthermore, the absolute configurations of the sugars were determined by gas chromatography according to a previously described method [24].

### 4.5. Cytotoxicity Assays

Cell Lines and Cell Culture

Hela, SGC-7901, and SHG-44 cancer cell lines were all obtained from the Chinese Academy of Medical Sciences Basic Medicine Cell Center (Beijing, China). Cells were cultured in DMEM media containing 10% FBS and 1% penicillin/streptomycin in a 37 °C humidified incubator with 5% CO_2._

Cell Viability Assay

A cell viability assay was conducted using the MTT method according to manufacturer’s protocol. Briefly, cells cultured in 96-well plates at a density of 6 × 10^4^ cells/mL per well in a 96-well microtiter plate. Different concentrations of the isolated compounds dissolved in dimethyl sulfoxide (DMSO) were added to each well. Each concentration was tested in triplicate. After incubation at 37 °C in 5% CO_2_ for 48 h, 10 µL of MTT was added to each well, and incubation was continued for additional 4 h. Cell viability was quantified by reading the plates at an absorbance of 570 nm using a microplate reader.

## 5. Conclusions

Four new meroterpenoids, Clavilactone M-P (**1**–**4**), with an aminoglycoside moiety having a 10-membered carbocycle-fused hydroquinone, were obtained from the fruiting bodies of the basidiomycete *Clitocybeclavipes*. Their structures were determined by comprehensive analysis of spectroscopic data. All the isolated compounds (**1**–**4**) were evaluated for their antitumor activity against three human cancer cell lines (Hela, SGC-7901, and SHG-44) in vitro. Compounds **1** and **2** exhibited a significant suppression of cell viability in the Hela (IC_50_ = 22.8 and 19.7 μM) cell line.

## Figures and Tables

**Table 1:** NMR spectral data of **1**–**3** (600 MHz for ^1^H-NMR and 150 MHz for ^13^C-NMR in CD_3_OD).

No.	1		2		3	
	δ_H_(*J* in Hz)	δc	δ_H_(*J* in Hz)	δc	δ_H_(*J* in Hz)	δc
1	-	186.1	-	185.8	-	186.1
2	5.94(s)	103.0	6.27(s)	103.8	-	148.1
3	-	148.1	-	147.5	5.92(s)	102.7
4	-	183.0	-	182.8	-	183.0
5	-	151.9	-	151.8	-	146.1
6	5.98(s)	74.1	5.97(s)	73.5	5.94(s)	74.0
7	4.24(s)	64.2	4.23(s)	64.1	4.24(s)	64.3
8	-	61.8	-	61.6	-	61.9
9	1.30(m), 2.60(d,13.2),	25.7	1.29(m), 2.58(m),	25.5	1.30(m), 2.60(d,12.0),	25.7
10	2.18(m), 2.35(m),	23.8	2.21(m), 2.35(m),	23.6	2.22(m), 2.35(m),	23.8
11	5.34(br, s)	124.9	5.33(br, s)	124.7	5.34(br, s)	125.2
12	-	136.9	-	136.7	-	139.0
13	2.84(d,12.0), 3.67(d,12.0),	27.8	2.84(d,14.4), 3.69(d,14.4),	27.6	2.84(d,12.0), 3.67(d,12.0),	27.5
14	-	135.6	-	135.5	-	136.4
15	1.53(3H, s)	23.4	1.41(3H, s)	23.1	1.53(3H, s)	23.2
16	-	173.8	-	173.6	-	173.8
1′	4.52(d,8.4)	84.4	5.11(d,4.8)	82.3	4.53(d,8.4)	84.3
2′	3.35~3.70 (4H, m)	74.1	3.35–3.76 (4H, m)	74.0	3.4~3.70 (4H, m)	74.1
3′		78.8		78.6		78.8
4′		71.5		71.6		71.5
5′		79.6		79.4		79.6
6′	3.68(dd, 2.4, 12.0) 3.86(dd, 5.4, 12.0)	62.7	3.68(m) 3.73(m)	62.3	3.68(dd, 5.4, 12.0) 3.86(dd, 2.4, 12.0)	62.7

s = singlet; d = doublet; t = triplet; m = multiplet; dd = doublet of doublet; br, s = broad singlet.

**Table 2:** NMR spectral data of **4** (600 MHz for ^1^H-NMR and 150 MHz for ^13^C-NMR in DMSO-d6).

No.	δ_H_(*J* in Hz)	δc	No.	δ_H_(*J* in Hz)	δc
1	-	183.9	12	-	134.1
2	-	149.2	13	2.66(d,12.0) 3.57(d,12.0)	26.6
3	-	146.9			
4	-	181.9	14	-	135.1
5	-	133.3	15	1.47(3H, s)	21.8
6	5.87(s)	72.7	16	-	172.1
7	4.5(s)	62.9	1′	4.39(t, 7.2)	83.1
8	-	60.6	2′	3.10–3.41 (4H, m)	78.6
9	2.54(br, s) 1.30(m)	24.3	3′		77.2
			4′		72.1
10	2.19(br, s) 2.08(m)	22.5	5′		70.2
			6′	3.66(dd, 4.2, 12.0) 3.42(m)	61.2
11	5.29(br, s)	123.4			

**Table 3:** In vitro cytotoxic activity of compounds **1**–**4**.

Compounds	IC_50_ (µM)		
	Hela	SGC-7901	SHG-44
**1**	22.8 ± 0.9	53.5 ± 1.2	>100
**2**	19.7 ± 1.1	38.4 ± 0.8	29.5 ± 0.8
**3**	55.2 ± 0.9	34.8 ± 1.3	35.8 ± 1.0
**4**	44.5 ± 0.7	47.9 ± 1.6	>100
Cisplatin	2.4 ± 0.02	2.0 ± 0.04	1.5 ± 0.03

## Data Availability

All data are available in the main text or the Supplementary Materials.

## Supplementary Materials

The following supporting information can be downloaded at: https://www.mdpi.com/article/10.3390/molecules28145456/s1.

## Abbreviations

The following abbreviations are used in this manuscript:

IR	Infrared
NMR	Nuclear magnetic resonance
HR-ESI-MS	High resolution electrospray ionization mass spectroscopy
HMBC	Heteronuclear multiple bond correlation
HSQC	Heteronuclear single quantum correlation
COSY	Homonuclear chemical shift Correlation Spectroscopy
NOESY	Nuclear Overhauser effect spectroscopy
TMS	Tetramethylsilane
ODS	Octadecyl silane
HPLC	High performance liquid chromatography
CH_2_Cl_2_	Dichloromethane
EtOAc	Ethyl acetate
MeOH	Methanol
MTT	3-(4,5-Dimethylthiazol-2-yl)-2,5-diphenyltetrazolium bromide
