# Supplementary material for: Four Meroterpenoids with Novel Aminoglycoside Moiety from the Basidiomycete Clitocybe clavipes with Cytotoxic Activity
Source: Molecules. 2023 Jul 17;28(14):5456. doi: 10.3390/molecules28145456 (PMC10384625; doi:10.3390/molecules28145456)
Supplement: Supplementary file 1 [file molecules-28-05456-s001.zip › molecules-2495335-supplementary.pdf]

Four meroterpenoids with novel aminoglycoside moiety from the Basidiomycete *Clitocybe clavipes* with Cytotoxic Activity

Sun Zhonghao [1,†], Ma Yongben [2,†], Zhang Jiawen [1], Ma Guoxu [1], Wu Haifeng [1], Shi Leiling [3], Sun Zhaocui [1*] and Xu Xudong [1*]

[1] These authors contributed equally to this work.

[1] Institute of Medicinal Plant Development, Chinese Academy of Medical Sciences & Peking Union Medical College, Beijing 100193, China; sun_zhonghao@126.com (S.Z.); zjwen00071@163.com (Z.J.); gxma@implad.ac.cn (M.G.); hfwu@implad.ac.cn (W.H.)

[2] School of Pharmaceutical Sciences, Hebei University of Chinese Medicine, Shijiazhuang 050091, China; Mayongben@hebcm.edu.cn

[3] Xinjiang Institute of Chinese and Ethnic Medicine, Urumqi 830002, China; shileiling@sina.com

* Correspondence: flydancingsun@163.com (S.Z.); xdxu@implad.ac.cn (X.X.)

† These authors contributed equally to this work.

Commented [M1]: Please carefully check the accuracy of names and affiliations.

Commented [M2]: Please add the affiliation numbers.

Commented [M3]: The name of this author is different from the one submitted online at susy.mdpi.com. Please check and confirm the fist names and last names.

Commented [M4]: We added the email addresses here according to those submitted online at susy.mdpi.com. Please confirm.

Commented [M5]: For universities, the department/school/faculty/campus is required. Please try to provide this information.

Abstract: Four new meroterpenoids, Clavilactone M-P (**1**–**4**) with novel aminoglycoside moiety having a 10-membered carbocycle connected to a hydroquinone and an α, β-epoxy/unsaturated lactone, were obtained from the fruiting bodies of the basidiomycete *Clitocybe clavipes*. Their structures were determined by comprehensive analysis of their spectroscopic data. All the isolated compounds (**1**–**4**) were tested for their cytotoxic activity against three human tumor cell lines (Hela, SGC-7901, and SHG-44) in vitro after treatment for 48 h. Compound **1** and **2** exhibited a signi ficant suppression of cell viability in the Hela (IC50 = 22.8 and 19.7 μM) cell line.

Keywords: meroterpenoid; Clavilactone; *Clitocybe clavipes*; cytotoxicity

List of Figures S1-S34

Figure S1. ^{1}H-NMR (600 MHz, CD_3OD) spectrum of the new compound **1**

Figure S2. ^{13}C-APT (150 MHz, CD_3OD) spectrum of the new compound **1**

Figure S3. ^{1}H-^{1}H COSY spectrum of the new compound **1**

Figure S4. HSQC spectrum of the new compound **1**

Figure S5. HMBC spectrum of the new compound **1**

Figure S6. ROESY spectrum of the new compound **1**

Figure S7. HR-ESI-MS spectrum of the new compound **1**

Figure S8. ^{1}H-NMR (600 MHz, CD_3OD) spectrum of the new compound **2**

Figure S9. ^{13}C-APT (150 MHz, CD_3OD) spectrum of the new compound **2**

Figure S10. ^{1}H-^{1}H COSY spectrum of the new compound **2**

Figure S11. HSQC spectrum of the new compound **2**

Figure S12. HMBC spectrum of the new compound **2**

Figure S13. ROESY spectrum of the new compoikund **2**

Figure S14. HR-ESI-MS spectrum of the new compound **2**

Figure S15. ^{1}H-NMR (600 MHz, CD_3OD) spectrum of the new compound **3**

Figure S16. ^{13}C-APT (150 MHz, CD_3OD) spectrum of the new compound **3**

Figure S17. ^{1}H-^{1}H COSY spectrum of the new compound **3**

Figure S18. HSQC spectrum of the new compound **3**

Figure S19. HMBC spectrum of the new compound **3**

Figure S20. ROESY spectrum of the new compound **3**

Figure S21. HR-ESI-MS spectrum of the new compound **3**

Figure S22. ^{1}H-NMR (600 MHz, DMSO-d6) spectrum of the new compound **4**

Figure S23. ^{13}C-APT (150 MHz, DMSO-d6) spectrum of the new compound **4**

Figure S24. ^{1}H-^{1}H COSY spectrum of the new compound **4**

Figure S25. HSQC spectrum of the new compound **4**

Figure S26. HMBC spectrum of the new compound **4**

Figure S27. ROESY spectrum of the new compound **4**

Figure S28. HR-ESI-MS spectrum of the new compound **4**

Figure S29. experimental electronic circular dichroism (ECD) spectra of **1-4**

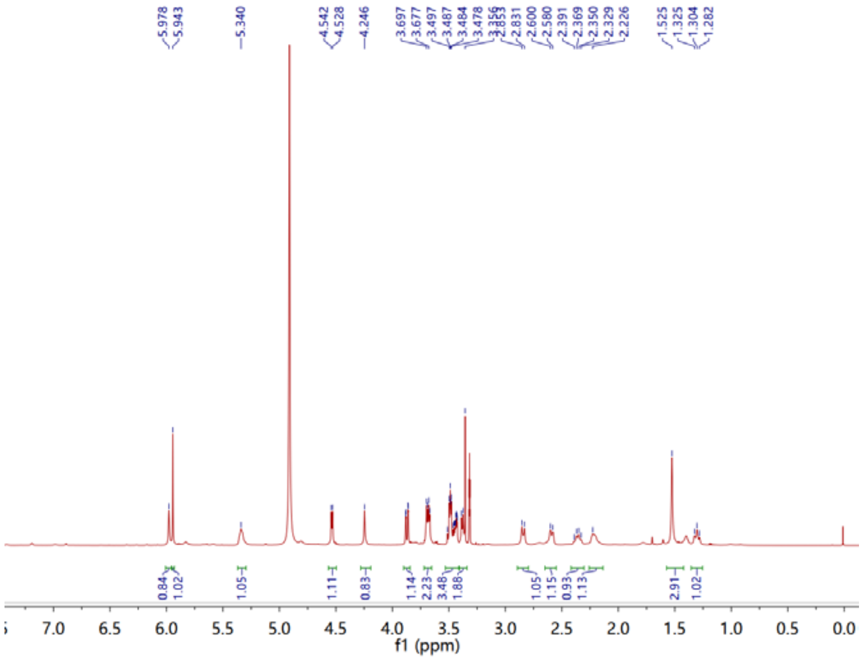

Figure S1. ^{1}H-NMR (600 MHz, CD_3OD) spectrum of the new compound **1**

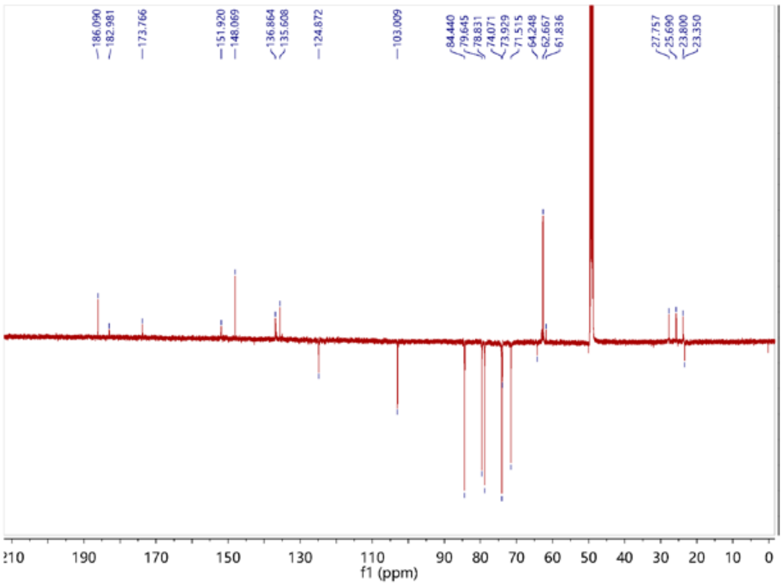

Figure S2. ^{13}C-APT (600 MHz, CD_3OD) spectrum of the new compound **1**

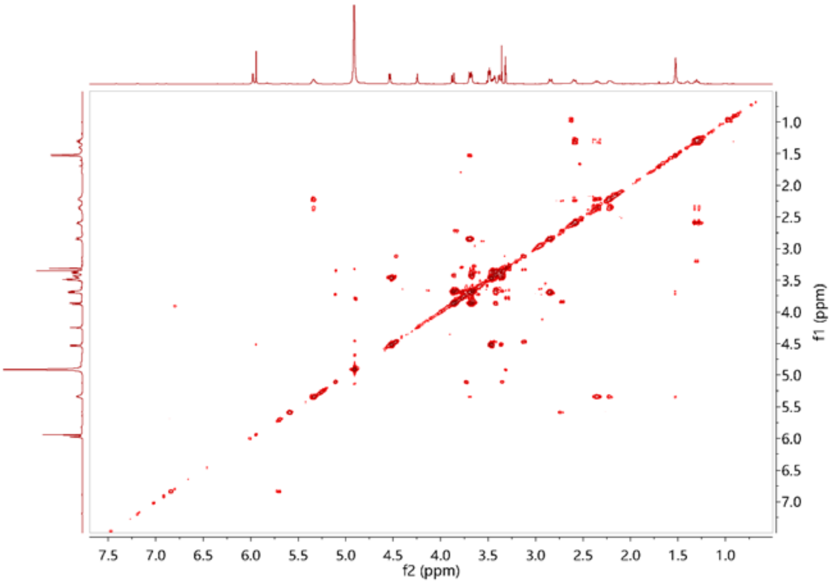

Figure S3. ^{1}H-^{1}H COSY spectrum of the new compound **1**

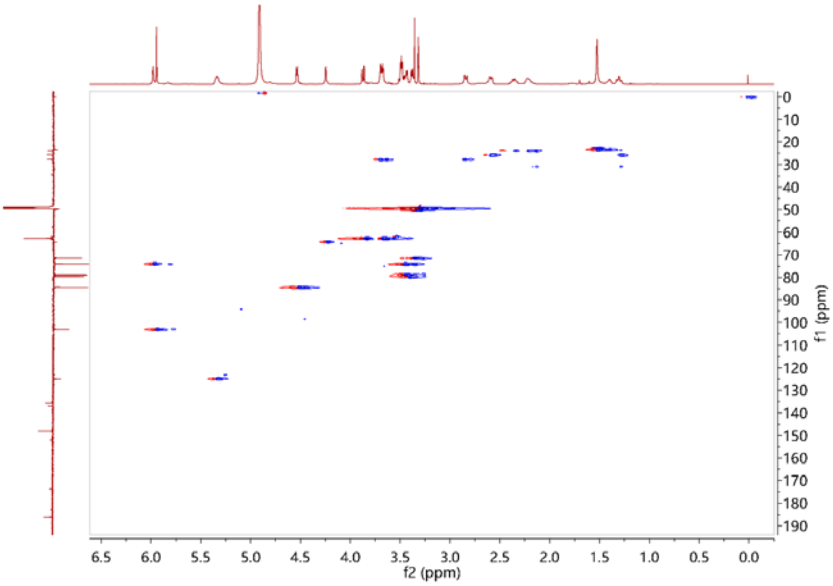

Figure S4. HSQC spectrum of the new compound **1**

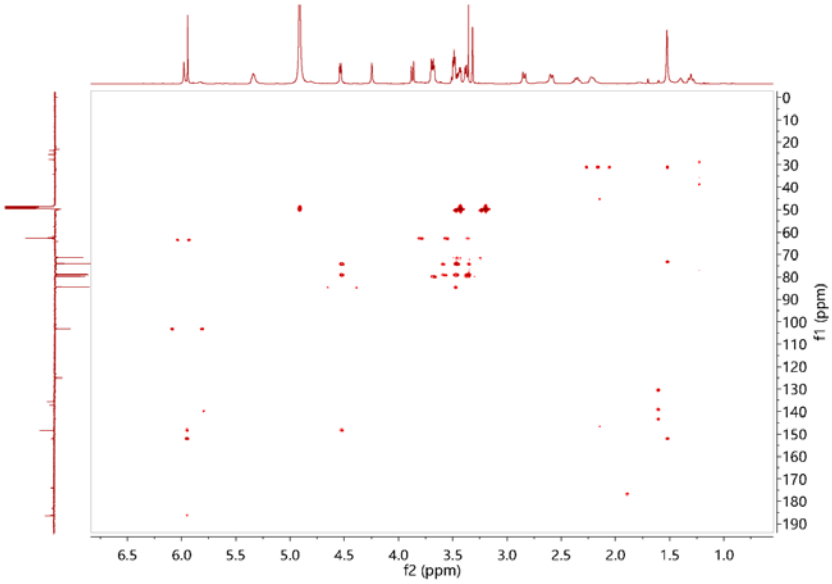

Figure S5. HMBCspectrum of the new compound **1**

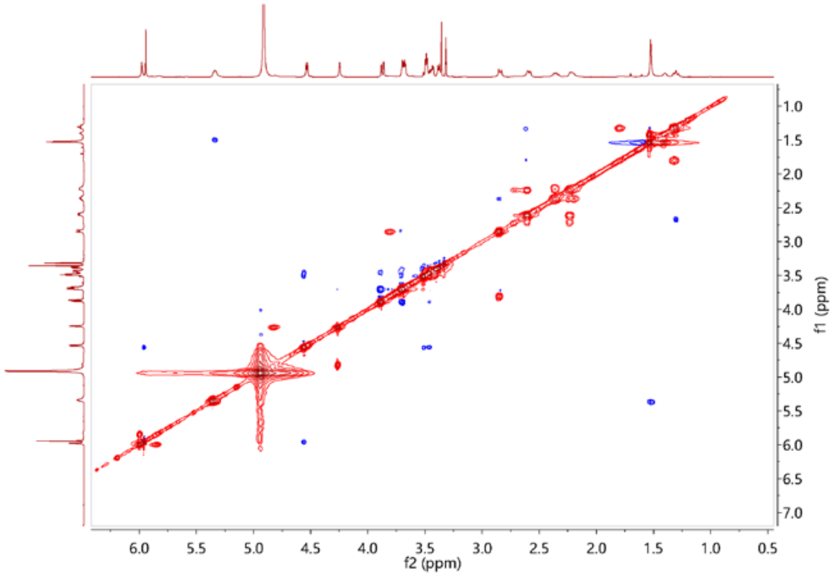

Figure S6. ROESY spectrum of the new compound **1**

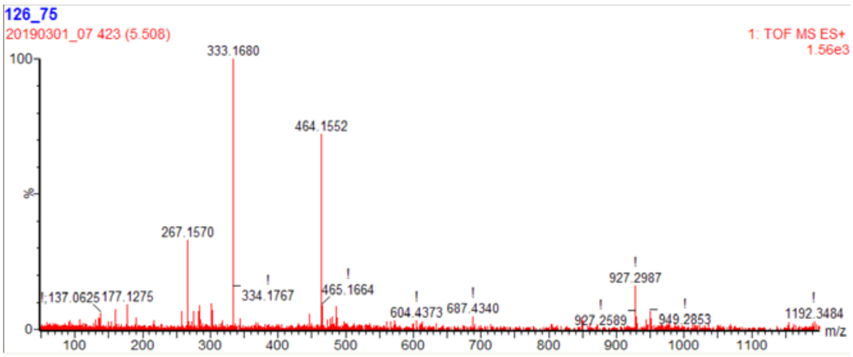

Figure S7. HR-ESI-MS spectrum of the new compound **1**

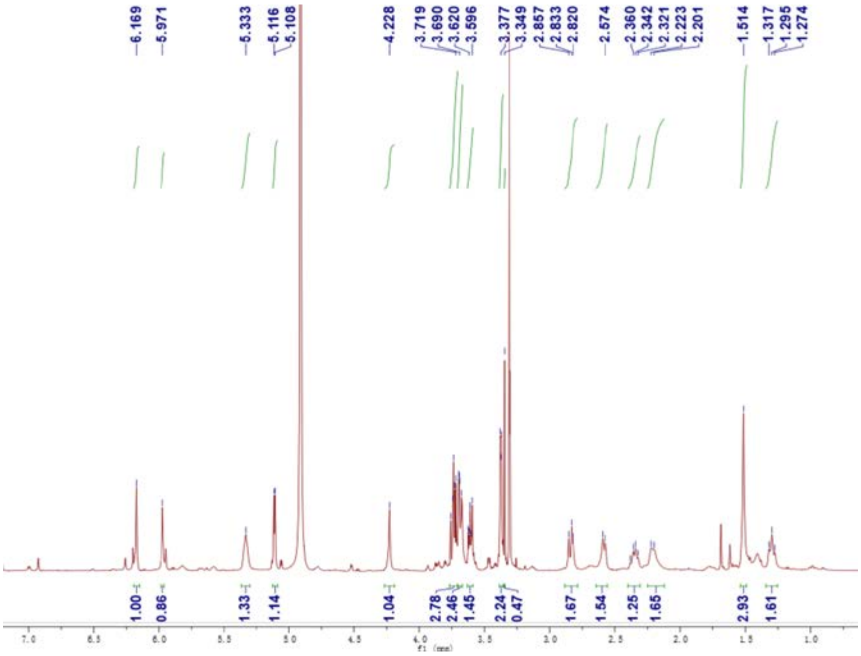

Figure S8. ^{1}H-NMR (600 MHz, CD_3OD) spectrum of the new compound **2**

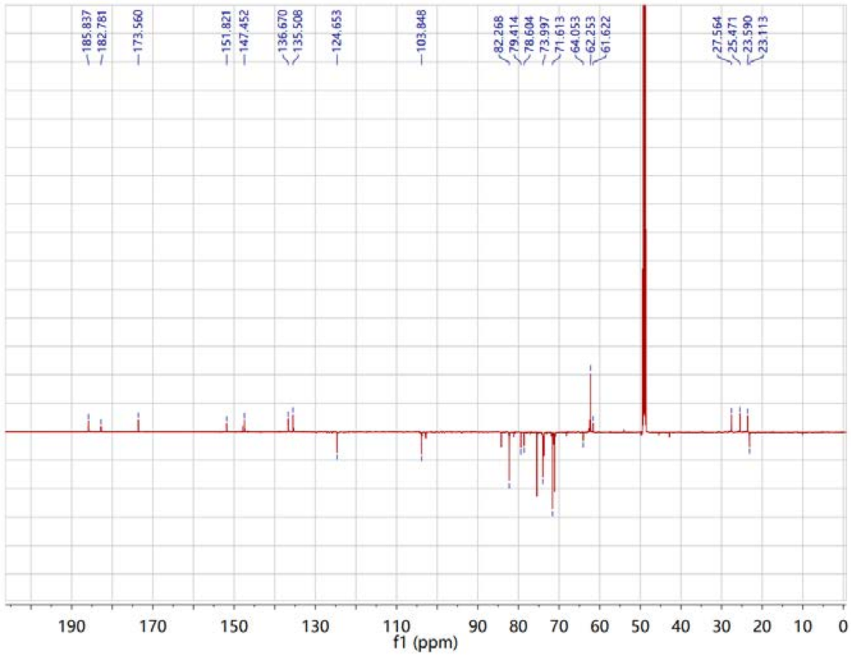

Figure S9. ^{13}C-APT (150 MHz, CD_3OD) spectrum of the new compound **2**

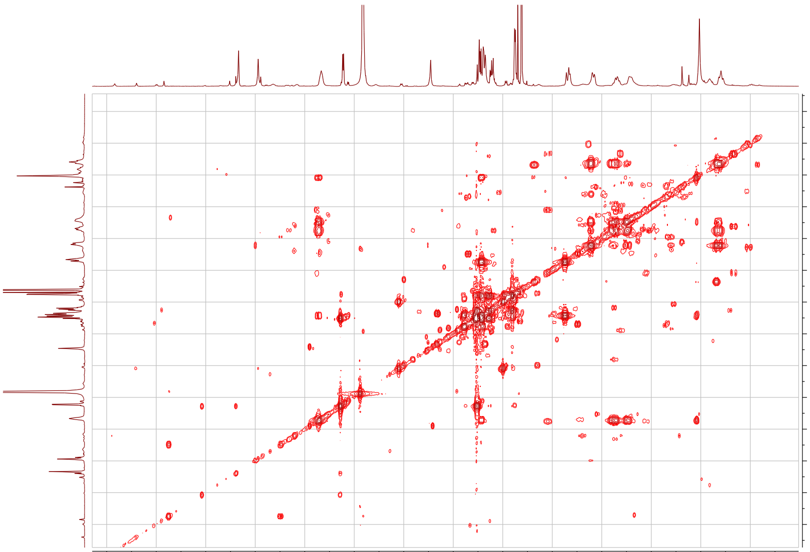

Figure S10. ^{1}H-^{1}H COSY spectrum of the new compound **2**

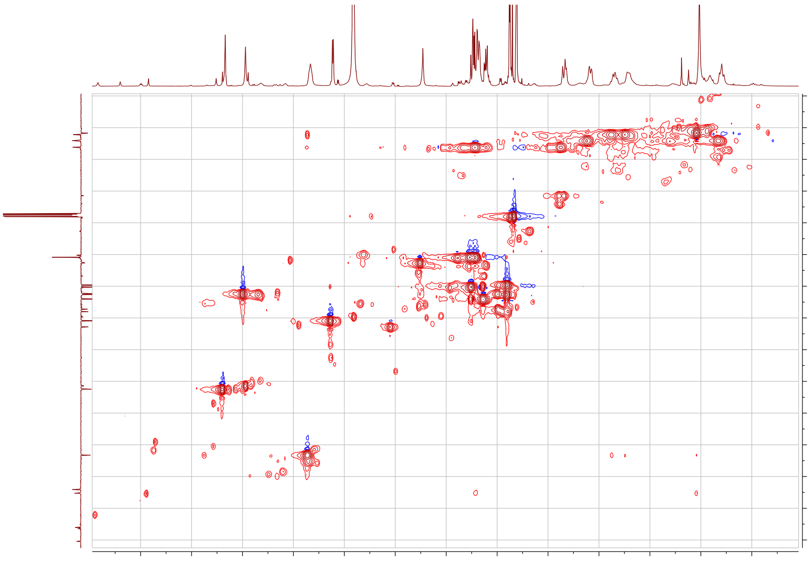

Figure S11. HSQC spectrum of the new compound **2**

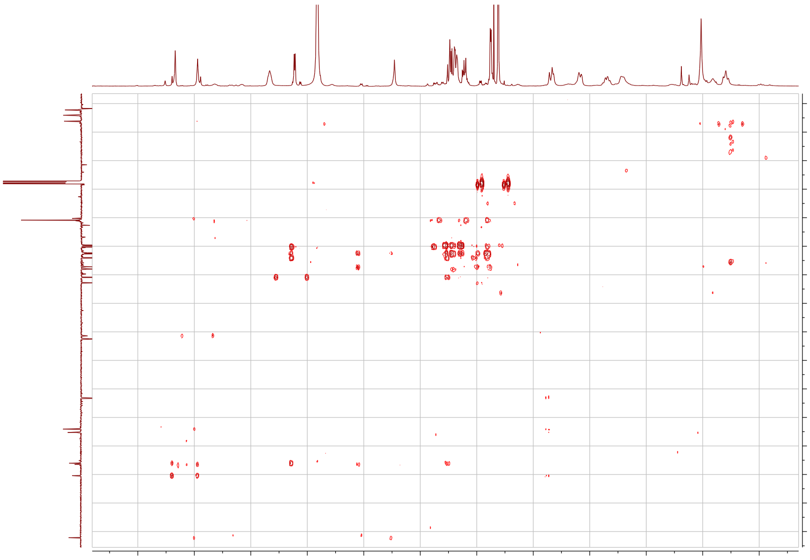

Figure S12. HMBC spectrum of the new compound **2**

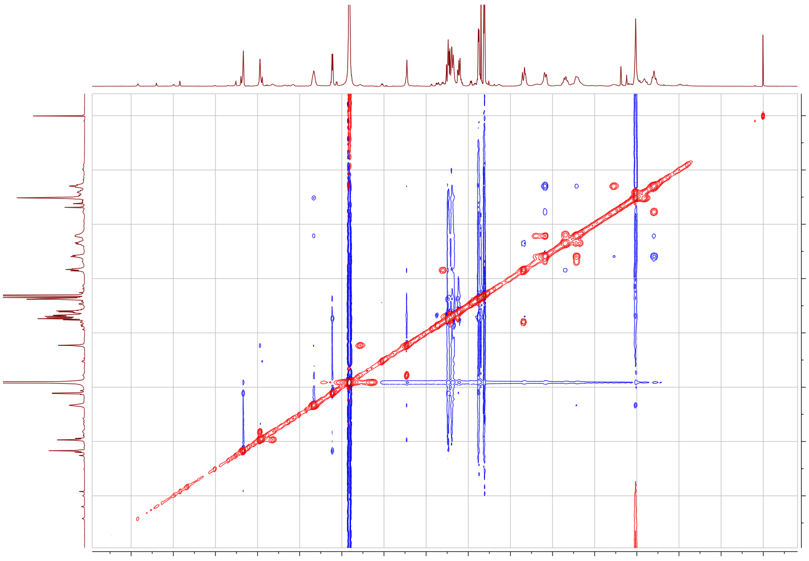

Figure S13. ROESY spectrum of the new compound **2**

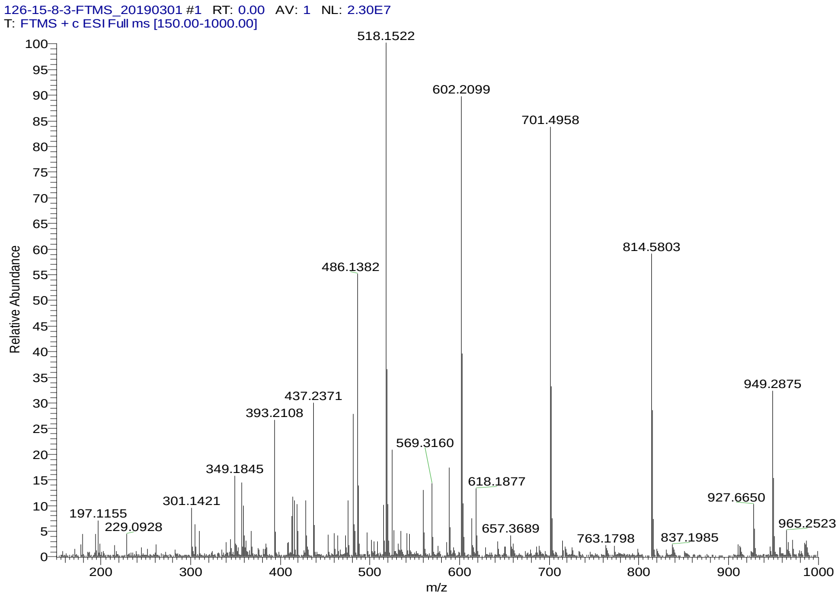

Figure S14. HR-ESI-MS spectrum of the new compound **2**

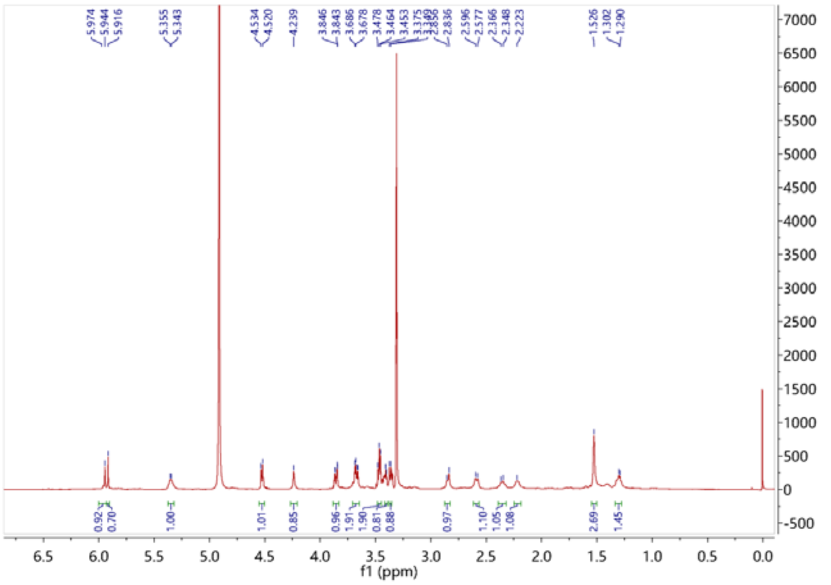

Figure S15. ^{1}H-NMR (600 MHz, CD_3OD) spectrum of the new compound **3**

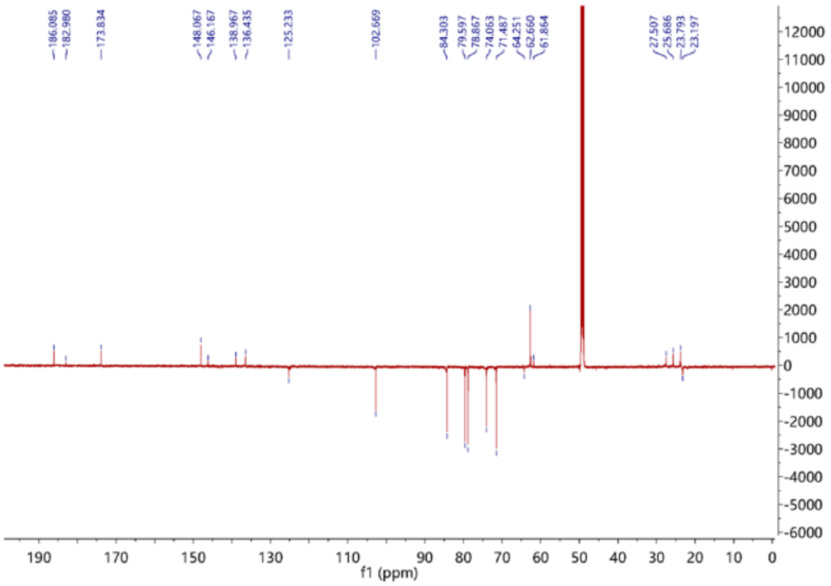

Figure S16. ^{13}C-APT (150 MHz, CD_3OD) spectrum of the new compound **3**

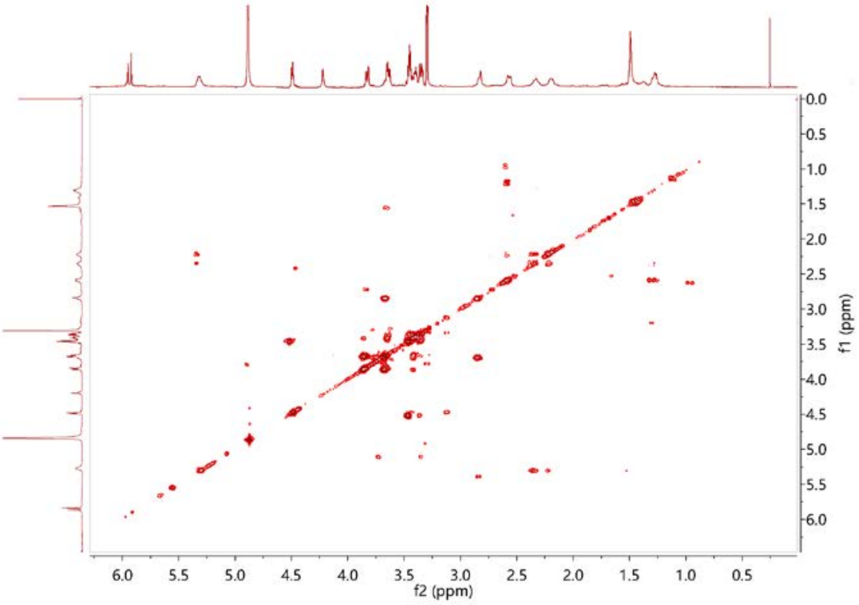

Figure S17. ^{1}H-^{1}H COSY spectrum of the new compound **3**

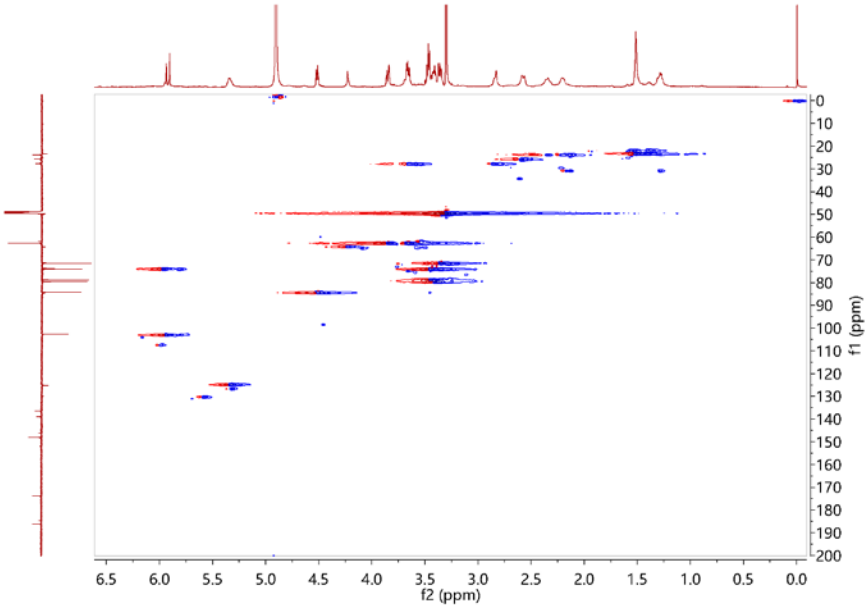

Figure S18. HSQC spectrum of the new compound **3**

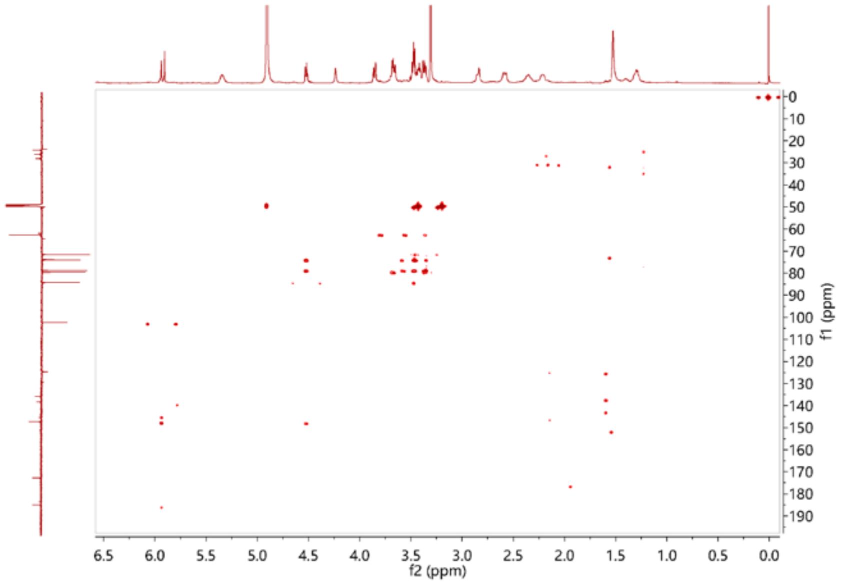

Figure S19. HMBC spectrum of the new compound **3**

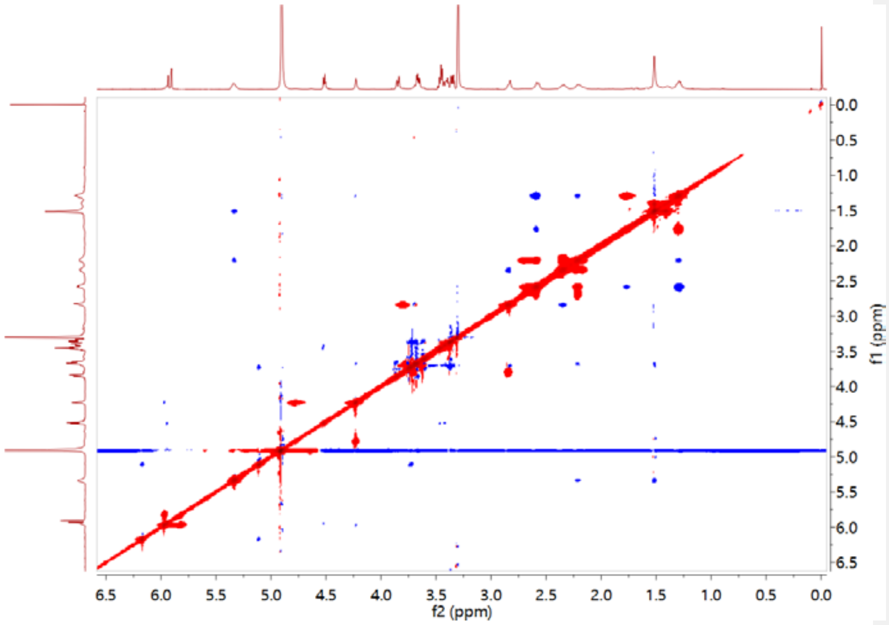

Figure S20. ROESY spectrum of the new compound **3**

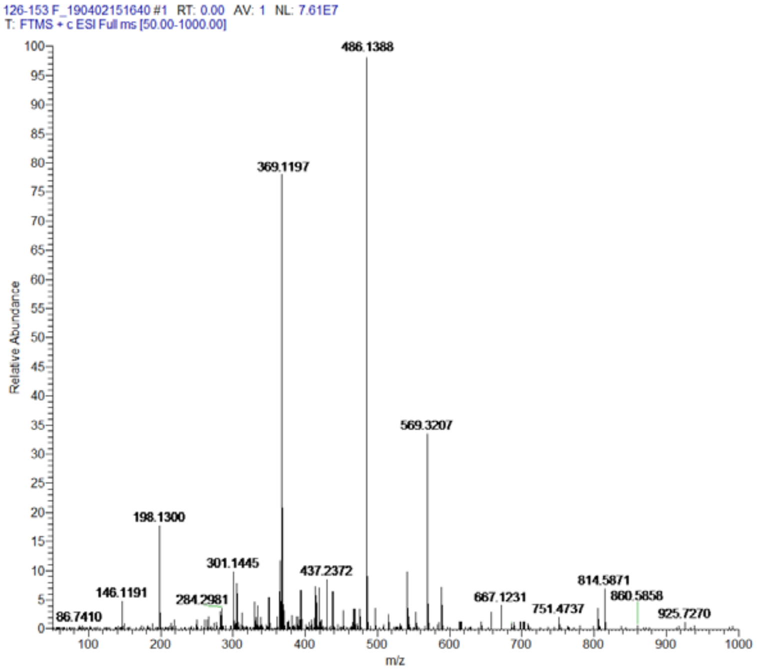

Figure S21. HR-ESI-MS spectrum of the new compound **3**

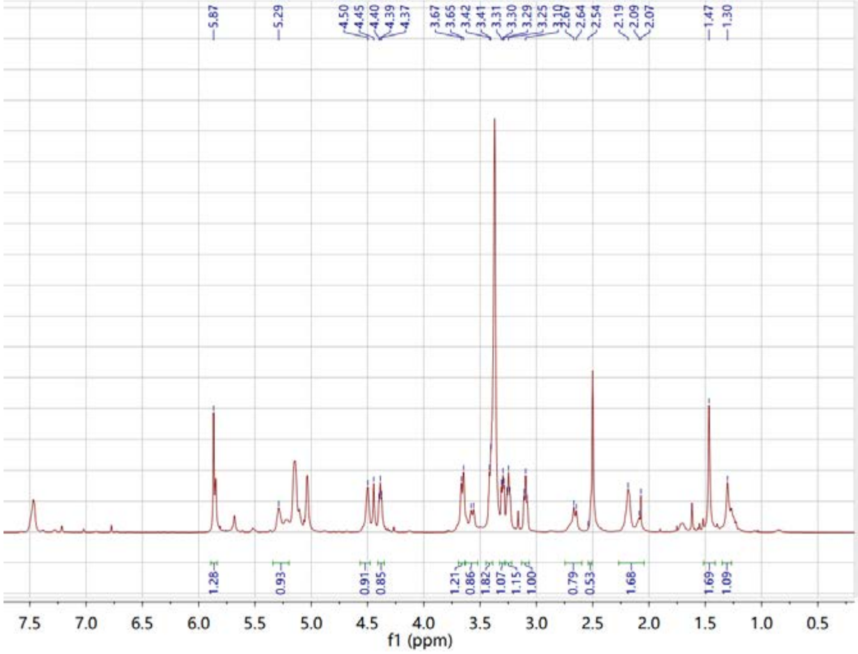

Figure S22. ^{1}H-NMR (600 MHz, DMSO-d6) spectrum of the new compound **4**

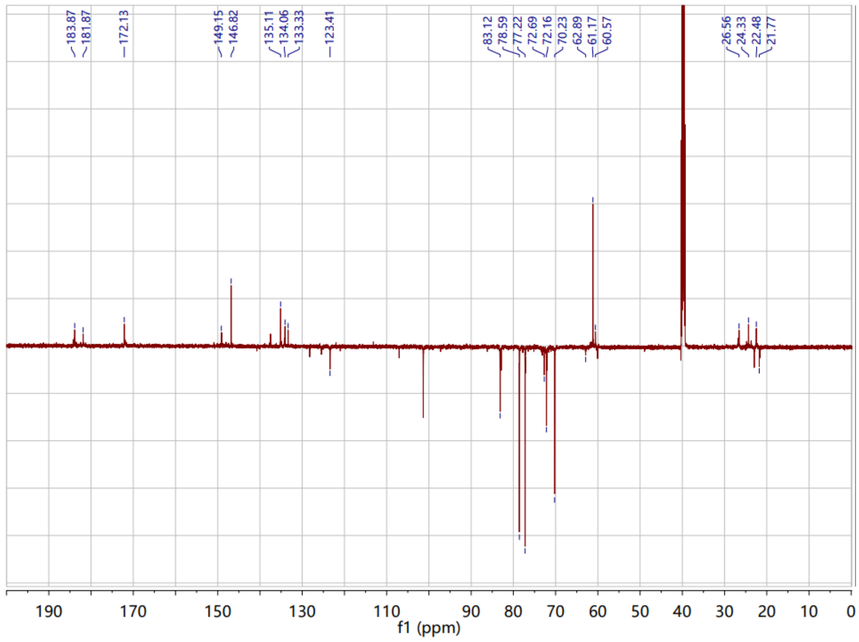

Figure S23. ^{13}C-APT (150 MHz, DMSO-d6) spectrum of the new compound **4**

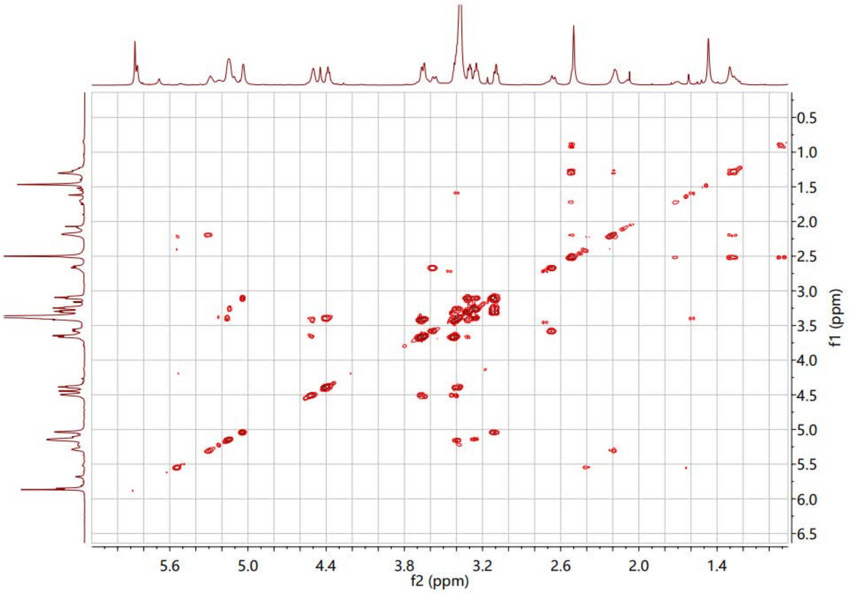

Figure S24. ^{1}H-^{1}H COSY spectrum of the new compound **4**

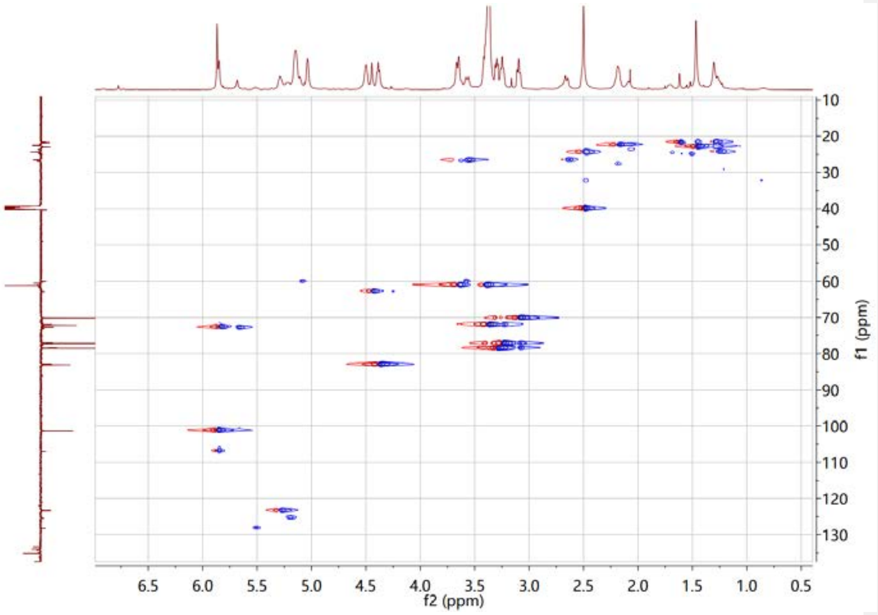

Figure S25. HSQC spectrum of the new compound **4**

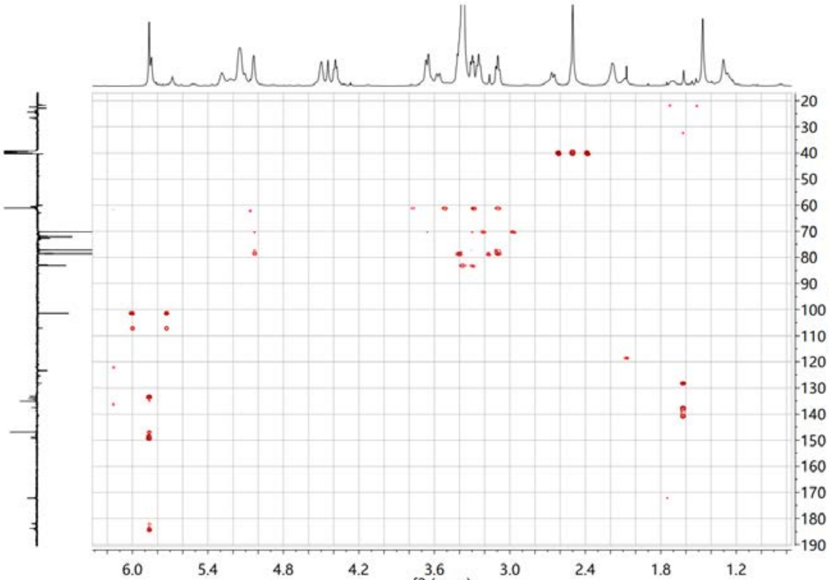

Figure S26. HMBC spectrum of the new compound **4**

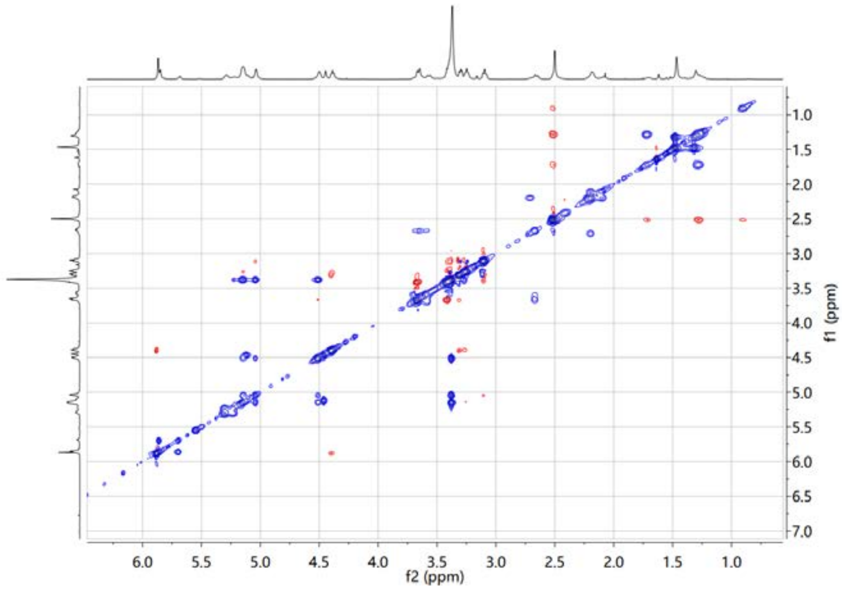

Figure S27. ROESY spectrum of the new compound **4**

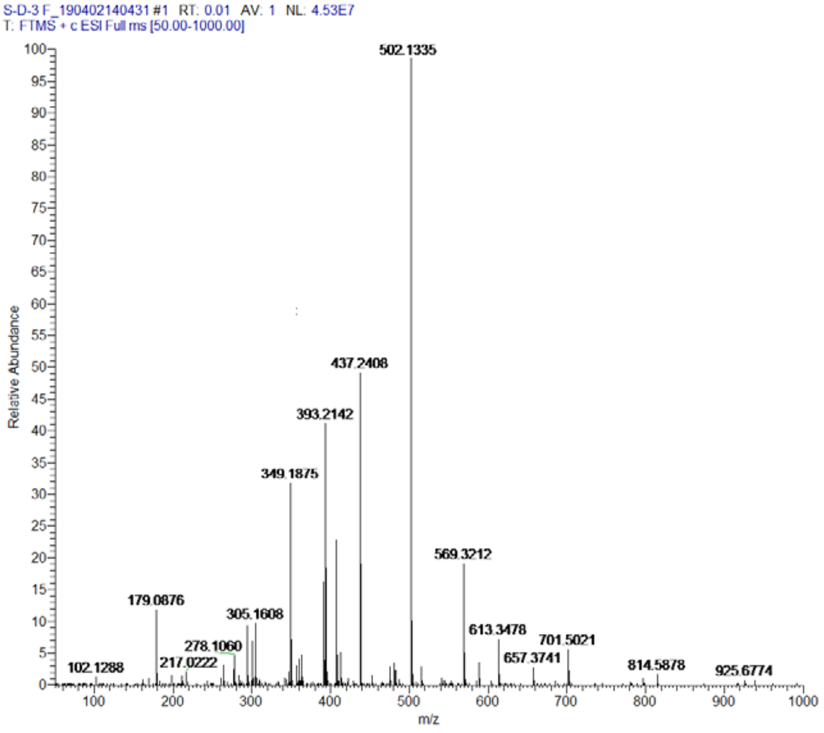

Figure S28. HR-ESI-MS spectrum of the new compound **4**

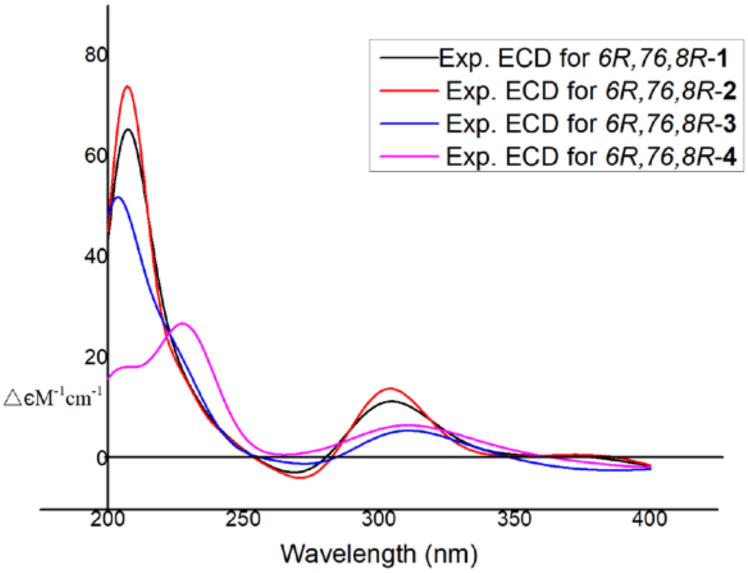

Figure S29. experimental electronic circular dichroism (ECD) spectra of **1-4**
